# 慢性中性粒细胞白血病转化为急性淋巴细胞白血病1例

**DOI:** 10.3760/cma.j.cn121090-20250429-00204

**Published:** 2026-01

**Authors:** 岩 尉, 亚哲 王, 文冰 段, 倩 江

**Affiliations:** 北京大学人民医院，北京大学血液病研究所，国家血液系统疾病临床医学研究中心，北京 100044 Peking University People's Hospital, Peking University Institute of Hematology, National Clinical Research Center for Hematology Disease, Beijing 100044, China

患者，男，29岁。2017年5月初常规体检发现WBC 30×10^9^/L，无任何不适。2017年5月27日外院就诊行系统检查。血常规：WBC 39.22×10^9^/L，HGB 145 g/L，PLT 232×10^9^/L。外周血白细胞分类以中性粒细胞为主，晚幼粒细胞3％，杆状核粒细胞38％，分叶核粒细胞45％（图1A）。骨髓细胞形态提示增生极度活跃，粒∶红为11.27∶1。粒系比例明显增高，胞质颗粒增多、增粗，早幼粒细胞3.0％，中幼粒细胞24.5％，晚幼粒细胞24.0％，杆状核粒细胞18.5％，分叶核粒细胞13.5％（图1B）。骨髓活检：造血细胞散在或灶性分布，粒系比例增高，未见原始细胞及异常淋巴细胞明显增多。网状纤维染色MF-0级。二代测序检测（NGS）：CSF3R存在2个位点突变：p.M617_T618delinsVI，等位基因突变频率（VAF）为41.2％；p.Y752*，VAF为42.7％；未检测到JAK2、CALR、MPL及SETBP1突变。染色体核型为46,XY[20]。腹部超声：脾大。诊断慢性中性粒细胞白血病（CNL）。

**图1:**
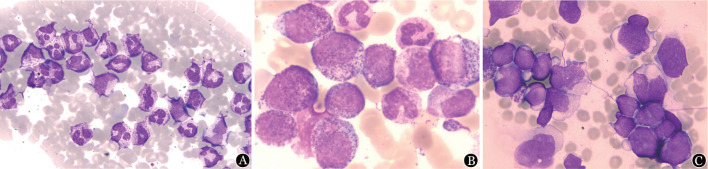
患者诊断慢性中性粒细胞白血病时外周血（A）、骨髓细胞形态（B）及转化为急性淋巴细胞白血病时骨髓细胞形态（C）

自2017年6月至2018年9月，患者每3～6个月监测血常规，未接受任何治疗，WBC波动于（28.7～33.8）×10^9^/L，分叶核+杆状核中性粒细胞比例波动于85％～95％。2018年9月3日，复查血常规：WBC 28.7×10^9^/L，HGB 134 g/L，PLT 232×10^9^/L。腹部超声：脾脏大小为18.2 cm×6.2 cm。患者因经济原因，接受羟基脲治疗。羟基脲起始剂量为1.0 g/d，随后根据WBC调整。服用羟基脲期间，WBC波动于（8.9～26.0）×10^9^/L。2021年1月23日复查血常规：WBC 24.8×10^9^/L，HGB 124 g/L，PLT 125×10^9^/L。腹部超声：脾脏大小为19.7 cm×7.3 cm。患者自此开始服用芦可替尼治疗（10 mg/次，每日2次），WBC波动于（14.2～33.8）×10^9^/L。自2022年2月起，患者出现盗汗及双下肢疼痛并逐渐加重，WBC逐渐升高。2022年2月24日腹部超声：脾脏大小为17.7 cm×6.1 cm。

2022年5月21日复查血常规：WBC 51.04×10^9^/L，HGB 110 g/L，PLT 152×10^9^/L。外周血白细胞分类：原始细胞占89％。中性粒细胞占9％，骨髓细胞形态提示增生明显活跃，原始淋巴细胞占有核细胞80％（图1C）。免疫分型：幼稚淋巴细胞占61.34％，表达CD34、CD19、CD38、CD123、HLA-DR、CD79a、CD13、CD58、TdT；部分表达CD33、CD22、CD20；不表达cCD3、CD7、cIgM、cKappa、cLambda，不除外急性B淋巴细胞白血病。B细胞来源的克隆性融合基因如BCR::ABL1、KMT2A、ZNF384及MEF2D均阴性。染色体核型：48, XY, add（1）（p36）, +8, +21[14]/46, XY[6]。NGS结果：CSF3R突变（p.M617_T618delinsVI, VAF 41.2％；p.Y752*, VAF 41.2％），BCORL1突变（p.Q1228fs, VAF 69.8％）。拷贝数变异分析（Copy number variant, CNV）：1号染色体短臂（chr1p36.22p36.33）部分缺失（该区域主要包括MTOR、PIK3D、GNB1等肿瘤相关基因）、1号染色体长臂部分重复（该区域主要包括AKT3等基因），+8（该区域主要包括MYC等基因），+21（该区域主要包括RUNX1等基因）。腹部超声：脾脏大小为16.3 cm×5.3 cm。该患者诊断急性淋巴细胞白血病（B细胞型，CNL转化）。患者接受了VDP方案（长春地辛、多柔比星、醋酸泼尼松）联合CD20单抗的诱导治疗，评估疗效达完全缓解。先后予Hyper-CVAD（B）、Hyper-CVAD（A）及贝林妥欧单抗巩固治疗，规律行腰椎穿刺并鞘内注射预防中枢神经系统白血病。2022年10月10日患者回输同胞全合供者外周血造血干细胞。目前无复发生存36个月，仅有轻微的慢性移植物抗宿主病（眼）。

讨论：CNL是一种罕见的骨髓增生性肿瘤，以成熟中性粒细胞持续性增多为特征，通常与CSF3R突变相关。中位发病年龄为66.5（15～86）岁，男性略多见（56％～58％）。10％～21％的CNL患者在中位21（3～94）个月内转化为急性髓系白血病。也有少数转化为真性红细胞增多症或慢性粒-单核细胞白血病。除了CSF3R突变外，也可见到ASXL1、SETBP1、EZH2和U2AF1等基因突变，但生存期更短，转化为急性白血病的风险更高。

CNL转化为急性淋巴细胞白血病的病例非常少见。Craig等报道了1例80岁CNL患者，予芦可替尼联合羟基脲治疗15个月后转为急性淋巴细胞白血病（B细胞型）。该患者疾病发生转化时，伴CSF3R T618I、ASXL1、SRSF2及RUNX1突变。该患者在急性淋巴细胞白血病开始治疗前死于心肌梗死。Franziska报道了1例33岁男性CNL患者，经羟基脲、芦可替尼及聚乙二醇干扰素治疗后，转化为急性混合细胞白血病（B细胞和髓系细胞）。其接受了同胞全合异基因造血干细胞移植（allo-HSCT），移植后24个月仍然生存。该患者在疾病转化阶段伴有CSF3R T618I、CSF3R W791*截短突变（胚系）、ASXL1 C759fs突变及新获得的RUNX1 R201Q突变。推断RUNX1突变促使了RUNX1相关的克隆转化。

本例患者在疾病转化阶段除伴有较为特异的CSF3R T618突变、CSF3R胞质尾区的截短突变（Y752）外，抑制肿瘤生长的转录因子BCORL1及多个染色体片段也发生了改变，我们推断这些遗传学的改变促使该患者疾病进展。

CNL尚无统一的治疗规范。通过PLT、WBC及是否合并ASXL1突变识别为高危的可耐受移植的患者，除了接受芦可替尼、干扰素α、羟基脲或临床试验外，应尽早考虑allo-HSCT。我们报道的此例CNL患者，在转为急性淋巴细胞白血病经诱导巩固治疗后成功序贯allo-HSCT，并且移植后获得了长达36个月的无复发生存。本病例结合文献报道表明：CNL存在向淋系转化的潜能，对于转化后的能耐受移植的白血病患者，采用针对性的化疗联合allo-HSCT可能是实现长期生存的有效策略。

